# Increased vulnerability of COPD patient groups to urban climate in view of global warming

**DOI:** 10.2147/COPD.S174148

**Published:** 2018-10-23

**Authors:** Christina Hoffmann, Marc Hanisch, Jana B Heinsohn, Vanessa Dostal, Melissa Jehn, Uta Liebers, Wulf Pankow, Gavin C Donaldson, Christian Witt

**Affiliations:** 1 Division of Ambulatory Pneumology, Charité – Universitätsmedizin Berlin, Berlin, Germany, christina.hoffmann2@charite.de; 2 Division of Pneumology and Infectiology – Thoracic Center, Vivantes Klinikum Neukölln, Berlin, Germany; 3 Airways Disease Section, National Heart and Lung Institute, Imperial College London, London, UK

**Keywords:** COPD, acute exacerbation, seasonal phenotype, urban heat island, heat stress, climate change

## Abstract

**Purpose:**

Patients with COPD show an increase in acute exacerbations (AECOPD) during the cold season as well as during heat waves in the summer months. Due to global climate changes, extreme weather conditions are likely to occur more frequently in the future. The goal of this study was to identify patient groups most at risk of exacerbations during the four seasons of the year and to determine at which temperature threshold the daily hospital admissions due to AECOPD increase during the summer.

**Patients and methods:**

We analyzed retrospective demographic and medical data of 990 patients, who were hospitalized for AECOPD in Berlin, Germany. The cases were grouped into the following cohorts: “spring” (admission between March and May), “summer” (June – August), “autumn” (September – November), and “winter” (December – February). AECOPD hospital admissions from 2006 and 2010 were grouped into a “hot summer” cohort and cases from 2011 and 2012 into a “cold summer” data-set. Climate data were obtained from the German Meteorological Office.

**Results:**

Patients hospitalized for a COPD exacerbation during winter were significantly older than summertime patients (*P*=0.040) and also thinner than patients exacerbating in spring (*P*=0.042). COPD exacerbations during hot summer periods happened more often to patients with a history of myocardial infarction (*P*=0.014) or active smokers (*P*=0.011). An AECOPD during colder summers occurred in patients with a higher Charlson index, who suffered in increased numbers from peripheral vascular diseases (*P*=0.016) or tumors (*P*=0.004). Summertime hospital admissions increased above a daily minimum temperature of 18.3°C (*P*=0.006).

**Conclusion:**

The identification of COPD patient groups most at risk for climate related exacerbations enables climate-adapted prevention through patient guidance and treatment. In view of global climate changes, discovering vulnerabilities and implementing adaptive measures will be of growing importance.

## Introduction

Patients with COPD show an increase in acute exacerbations (AECOPD) during the cold season^1^–^3^ as well as during heat waves in the summer months.^4^ A study by Mann et al found that higher mean outdoor temperatures, combined with a rise in barometric pressure, cause increased morning dyspnea in patients with COPD.^5^ Other studies have demonstrated an increase in hospital admissions due to exacerbations on days with high mean temperatures as well as high diurnal temperature ranges.^6^–^8^ Due to the global climate changes, extreme weather conditions are likely to occur more frequently in the future.^9^ It is, therefore, important to understand the vulnerability of COPD patients to climate.

The relationship between climate and mortality has already been investigated in several studies.^10^–^14^ For example, Zanobetti et al calculated an increased mortality hazard ratio for COPD patients aged over 65 years exposed to a high variability in summertime temperatures.^10^ The study focused on the standard deviation of summer temperatures, but did not discriminate between temperatures higher or lower than the average. Petkova et al analyzed urban heat-related mortality in New York City and concluded that demographic changes and the population’s adaptation to heat have a great influence on future mortality risk.^14^ They found a rapid adaptation to heat since the 1970s, possibly due to increased access to air conditioning.

It was the goal of our study to explore the relationship between climate and morbidity of COPD patients in an urban environment. As a measure for COPD morbidity, we chose hospitalization due to AECOPD.

The purpose of this study was three-fold:

Analyze the characteristics of patients whose admissions occurred during spring, summer, autumn, and winter to potentially identify a group most at risk of climate-related exacerbations, so that they could be targeted for adapted patient guidance and treatment,

Determine the temperature threshold at which daily hospital admissions due to AECOPD increase above normal during the summer,

Compare patient characteristics and AECOPD hospital admission rates between summers with outdoor temperatures above or below the decade’s average.

## Patients and methods

### Patients and data collection

**Figure 1:**
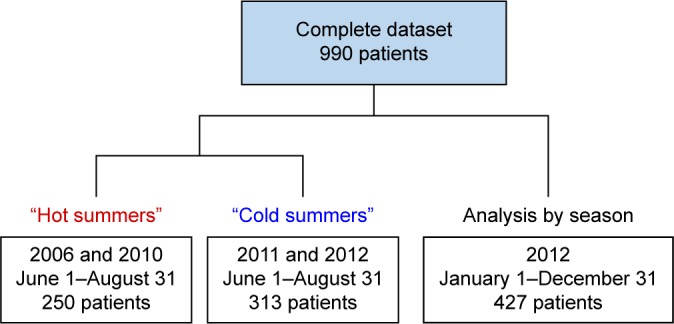
Study cohorts.

The study population was a consecutive sample of patients hospitalized for exacerbation of COPD, defined as International Classification Code-10 German Modification: J44.00-J44.99, with a main or secondary entry mentioning exacerbation. Demographic and medical data were retrospectively obtained from electronic patient records and paper notes of the Vivantes Hospitals in Berlin, Germany. The study was approved by the Ethics Committee of the Charité – Universitätsmedizin Berlin (EA1/199/13). Patients’ consent to retrospectively review their medical records was not required by the ethics committee, because section 25 of the Berlin Hospital Law (Berliner Landeskrankenhausgesetz) allows the usage of hospital patient data for research purposes after anonymization. All patient data were treated confidentially and anonymized prior to data analysis. The Charlson Index was used as a measure of the patients’ comorbidities.^15^,^16^ To avoid the issues caused by multiple statistical analyses run on the same dataset, we selected separate cohorts for the analysis of different summer periods and seasons of the year. The cohort for the comparison of hot and cold summer periods consisted of patients who visited the Vivantes Hospital in the district of Neukölln in 2006, 2010, 2011, and 2012. Patients for the analysis by season were admitted to the Vivantes Hospital in the Friedrichshain district in 2012. Figure 1 illustrates the time span and number of patients included in the different datasets.

### Environmental variables

Climate data on the daily minimum, mean, and maximum temperatures, air pressure, and air humidity were obtained from the meteorological station at Berlin-Tempelhof airport, available online through the website of the German Meteorological Office (Deutscher Wetterdienst, www.dwd.de). The meteorological station is located in the urban area of Berlin 48 m above the sea level at 52.47°N and 13.4°E.

### Statistical analysis

The statistical analysis was performed with IBM SPSS Statistics 25 (IBM Corporation, Armonk, NY, USA), and GraphPad Prism 7 (GraphPad Software Inc., La Jolla, CA, USA). As the date of symptom onset was unknown, we chose the hospital admission date as a measure of acute COPD exacerbation. A lag day analysis served to evaluate possible delays between symptom onset and hospitalization. Due to the anonymized patient data, multiple visits by the same patient occurred as separate cases in the datasets.

The AECOPD cases of the “analysis by season” dataset (see Figure 1) were grouped into the four cohorts “spring” (admission between March 1st and May 31st), “summer” (June 1st to August 31st), “autumn” (September 1st to November 30th), and “winter” (December 1st to February 29th). Between 2005 and 2014, the average temperature during the summer in Berlin, Germany was 19.2°C. The summer periods of 2006 and 2010 had hotter average temperatures (20.3°C, one sample *t*-test, *P*<0.001), while 2011 and 2012 featured colder summers (18.6°C, one sample *t*-test, *P*=0.009). We therefore grouped AECOPD hospital admissions from 2006 and 2010 in a “hot summer” cohort and cases from 2011 and 2012 in the “cold summer” group.

For statistical analysis, continuous variables of two groups were compared using Mann–Whitney *U* tests and nominal variables using Fisher’s exact tests or chi-squared tests. Comparisons between three or more groups were made using Kruskal-Wallis tests for continuous variables and with chi-square tests for nominal variables. Dunn’s multiple comparison test was used for post hoc tests. The relationship between hospital admissions per day due to AECOPD and climate variables was examined with Poisson regression analysis. Corresponding trajectories were calculated with a locally weighted scatter plot smoothing method (Loess), using a tri-weight function. This function allocates the least weight to outlying data points. A *P*-value below or equal to 0.05 was considered statistically significant.

## Results

### Characteristics of patients with exacerbations during spring, summer, autumn, and winter

**Figure 2:**
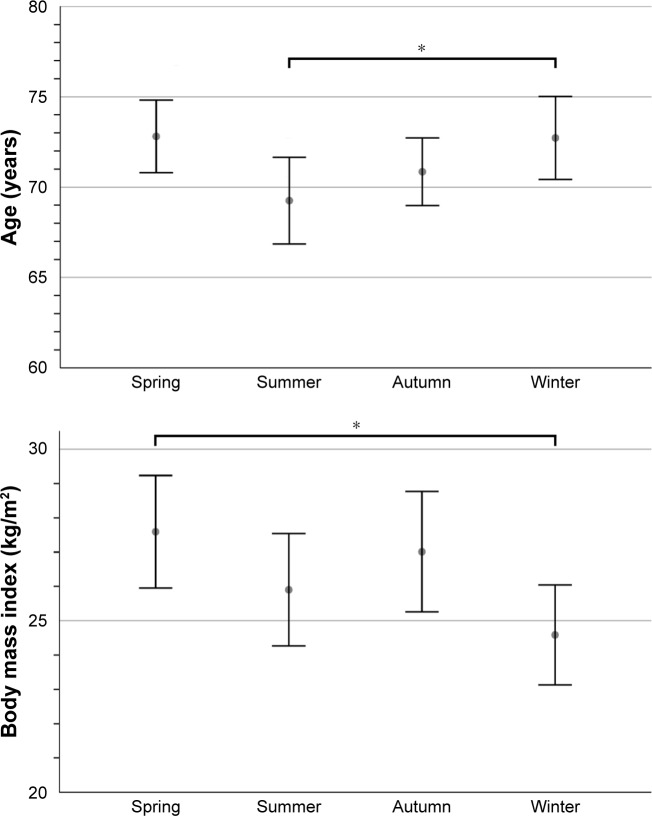
Differences in the age and BMI of patients with AECOPD in relation to the season. **Notes:** Displayed are mean values at 95% confidence intervals. ^*^P<0.05.

Table 1 summarizes the data collected for 427 patients who suffered from a COPD exacerbation, sorted by season. Kruskal-Wallis tests revealed significant differences between seasons in terms of patients’ age (*P*=0.018) and BMI (*P*=0.050). Figure 2 shows that patients with a COPD exacerbation during winter were significantly older than summertime patients (Dunn’s multiple comparison test, *P*=0.040). Patients hospitalized for AECOPD during winter were also significantly thinner than patients exacerbating in spring (Dunn’s multiple comparison test, *P*=0.042).

### Comparison of hospital admissions due to AECOPD between “hot” and “cold” summers

More patients with AECOPD were admitted to a hospital per day during the colder summers of 2011 and 2012 than in the hotter summers of 2006 and 2010 (Mann–Whitney *U* test, *P*=0.006). Table 2 lists the patient characteristics that were compared. We found significant differences in the patients’ Charlson index (Mann–Whitney *U* test, *P*=0.002) and smoking status (chi-squared test, *P*=0.016) between the hot and cold summer seasons. Table 3 illustrates the comorbidities present in more detail. Patients with COPD exacerbations during hot summer periods more often had a prior myocardial infarction (Fisher’s exact test, *P*=0.014). An AECOPD during colder summers occurred in patients with a higher Charlson index, in patients who were more likely to have a history of peripheral vascular diseases (Fisher’s exact test, *P*=0.016) or in patients with tumors (Fisher’s exact test, *P*=0.004). A closer inspection of the smoking status revealed that, especially in hot summers, more active smokers developed an AECOPD than former smokers (Fisher’s exact test, *P*=0.011). In 2006, which included the hottest summer period we investigated, 72% of the patients with a COPD exacerbation were active smokers, while only 18% were former smokers.

**Figure 3:**
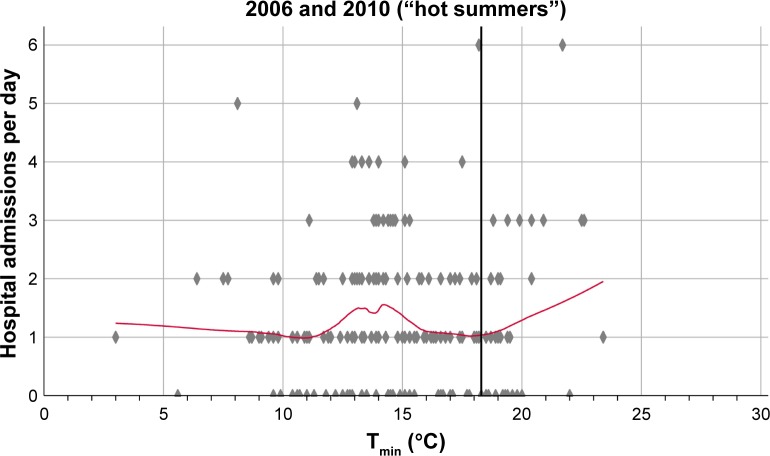

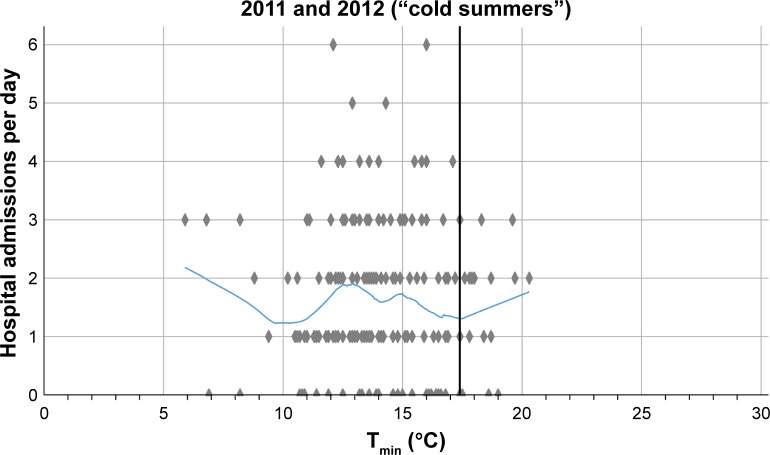
Increase in hospital admissions per day in relation to the minimum outdoor temperature. **Notes:** The number of hospital admissions per day due to AECOPD are displayed as diamond markings. The trajectories were calculated using a locally-weighted scatter plot smoothing method. A vertical line marks the threshold temperatures for increased hospital admissions during the summer as calculated by Poisson regression analysis. **Abbreviation:** T_min_, daily minimum outdoor temperature.

Figure 3 illustrates the results of a Poisson regression analysis. During hot summer seasons, hospital admissions per day due to AECOPD increased if the daily minimum outdoor temperature surpassed 18.3°C (Poisson regression analysis, *P*=0.006). In the colder summers, the best fit for a threshold temperature was 17.4°C, but did not reach statistical significance (Poisson regression analysis, *P*=0.322). The inclusion of other climate variables like air pressure and air humidity into the Poisson regression model resulted in no statistically significant improvement.

**Figure 4:**
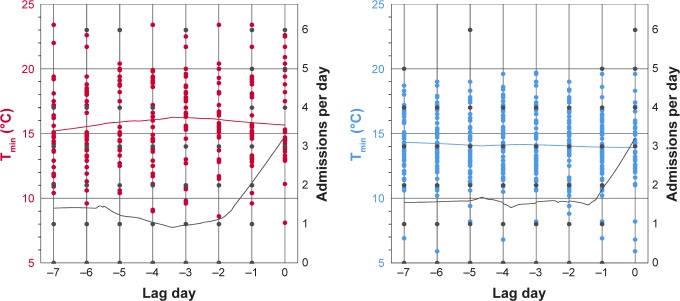
Temperature analysis on the days preceding an increase in hospital admissions. **Notes:** The figures show the daily minimum outdoor temperatures 1 week before an increase in hospital admissions to three or more AECOPD patients per day. The left column displays the results for hot summers, the right column for cold summers. Red and blue dots represent the recorded minimum outdoor temperatures. The number of admissions per day are shown as black dots. All trajectories were calculated with a locally-weighted scatter plot smoothing method. The black trajectories illustrate the daily admissions due to AECOPD, the red and blue curves represent minimum outdoor temperatures. **Abbreviation:** T_min_, daily minimum temperature.

Figure 4 shows an analysis of the outdoor temperatures for the 7 days before three or more hospital admissions per day occurred. Neither in the colder summers, nor in the hotter summers did the temperatures show a statistically significant difference on the days prior to an increase in hospital admissions.

## Discussion

Our findings that patients hospitalized for AECOPD during winter were significantly older and thinner than patients exacerbating in the other seasons could be influenced by hospitals reaching capacity limits. Increased patient loads, eg, during influenza epidemics, could influence emergency departments to preferentially admit only the older, frailer patients. We found no difference in GOLD stage distribution between the seasons (*P*=0.962) which suggests that the decision to admit a patient was clinically appropriate and unbiased by consideration of frailty. Our results are in accordance with a study from Tseng et al which found evidence that cold temperatures significantly affect the COPD exacerbation rate of elderly patients in Taiwan.^1^ Almagro et al also observed AECOPD patients in Barcelona, Spain to be older during winter.^17^ A possible explanation could be a lowered resilience to cold stress in elderly COPD patients. Holmér stated that the progressive degradation of physiological functions in elderly people increases the risk of harmful effects of cold exposure.^18^

Another result of our study was that more patients with AECOPD were admitted to an urban hospital per day during the colder summers we analyzed. This is in concordance with the observation of Gasparrini et al that milder but non-optimum weather contributes more to mortality risk than extreme temperatures.^12^ The adaptation capability of German COPD patients to heat stress could be modified in cold summer periods. Thermoregulatory response improves with repeated heat exposure and even passive exposure to heat, without any accompanying exercise, results in adaptation.^19^,^20^ Multiple heat waves during the hot summers could have improved the thermoregulatory response of the patients, leading to less hospital admissions than in the colder summers. An investigation of heat-related emergency hospitalization in the USA by Anderson et al revealed a significantly higher hospitalization risk in counties with cooler average summer temperatures. The association between temperature and hospital admissions was strongest on the same day, but still present 1 day after exposure.^6^ Similar to the results of Anderson et al, we found no statistically significant effect of lag days. This means that the delay between exposure to heat stress, occurrence of AECOPD, and hospital admission was not greater than 24 hours. For patients in Berlin, the capital of Germany, this seems feasible. Multiple hospitals are available within a short traveling distance of every inhabitant, with emergency departments open 24 hours every day.

Another explanation why the study did not reveal significant effects of lag days could be the limited amount of data from the 4 years. Time series analyses to detect lag day effects mostly investigate time spans of 10 years. For example, Scherer et al analyzed data from 2001 to 2010 and discovered that heat-stress related mortality in Berlin correlated best with the mean daily air temperature of five lag days.^11^ The limited power of our study could also be the reason why we did not find a threshold temperature with statistical significance for increased hospital admissions in colder summers. The daily minimum temperature in the cold summers of 2011 and 2012 surpassed the best fitting temperature threshold of 17.4°C only on 19 days. Nevertheless, the determined threshold for hot summer seasons, a daily minimum outdoor temperature of 18.3°C, can now be used to implement warning mechanisms for COPD patients.

In the cohorts we investigated, patients suffered more often from an AECOPD during hot summer periods if they had a history of myocardial infarction or were active smokers. An AECOPD during colder summers occurred more often in patients with a higher Charlson index, peripheral vascular disease, or tumor. These results are in accordance with previous studies. In a Dutch cohort, Westerik et al found associations between frequent COPD exacerbations and inter alia heart failure, pulmonary cancer, and peripheral vascular disease.^21^ A prospective cohort study in Spain revealed factors associated with frequent exacerbations to be inter alia the Charlson Index and smoking.^22^ During summer, the exposure to heat causes an increased cardiovascular strain.^23^ Patients with a myocardial infarction history or a peripheral vascular disease could be more vulnerable to a summertime AECOPD due to a reduced adaptive capacity to the increased cardiovascular stress. The adaptive capacity could be further limited by the vasoconstrictive effects of tobacco smoking.^24^ Patients with a tumor or a high Charlson Index could be more vulnerable through systemic weakening effects of their diseases.

The retrospective design and the usage of anonymized patient data put some limitations on the study results. Due to anonymization, multiple hospital admissions of the same patient occurred as separate cases in the dataset. This could potentially influence the results. Other potential confounders that were not analyzed are exposure to air pollutants, socioeconomic status, and possible fluctuations in the urban population due to seasonal travelings. To confirm and expand the results of our analysis, a prospective study, that includes cohorts with and without the seasonal AECOPD risk factors we have identified, should be performed.

## Conclusion

The identification of COPD patient groups most at risk of climate related exacerbations enables climate-adapted prevention through patient guidance and treatment. In view of the global climate changes, discovering vulnerabilities and implementing adaptive measures is of growing importance.

## Figures and Tables

**Table 1:** Comparison of patient characteristics and climate variables by season in the year 2012

Parameter	Spring	Summer	Autumn	Winter	Total	*P*-value
Admissions, n	120	90	109	108	427	-
Male, n (%)	61 (50.8%)	54 (60%)	72 (66.1%)	66 (61.1%)	253 (59.3%)	0.123
Female, n (%)	59 (49.2%)	36 (40%)	37 (33.9%)	42 (38.9%)	174 (40.7%)	0.123
Age, years, mean ± SD	72.8± 11.1	69.3± 11.5	70.9±9.9	72.7±12	71.4± 11.4	0.018*
BMI, kg/m^2^, mean ± SD	27.6±8	25.9±6.9	27.0±8.3	24.6±6.5	26.4±7.6	0.050*
Smoker, n (%)	37 (39.8%)	34 (44.7%)	40 (47.1%)	38 (46.3%)	149 (44.3%)	0.960
Former smoker, n (%)	52 (55.9%)	38 (50%)	41 (48.2%)	41 (50%)	172 (51.2%)	0.960
Non-smoker, n (%)	4 (4.3%)	4 (5.3%)	4 (4.7%)	3 (3.7%)	15 (4.5%)	0.960
Pack years, mean ± SD	43.4±27.4	43.3±23	46.9±28.2	42.2±28.5	44.1 ±26.8	0.633
FEV_1_, %, mean ± SD	36.6± 15.8	37.4±19.7	37.1 ± 15.6	37.2±16.3	39.5±16.9	0.937
GOLD I or II, n (%)	22 (20.8%)	17 (20.3%)	20 (20%)	16 (16.3%)	75 (19.3%)	0.889
GOLD III, n (%)	38 (35.8%)	24 (28.6%)	33 (33%)	36 (36.7%)	131 (33.8%)	0.889
GOLD IV, n (%)	46 (43.4%)	43 (51.2%)	47 (47%)	46 (46.9%)	182 (46.9%)	0.889
With LTOT, n (%)	40 (71.4%)	24 (63.2%)	40 (58.8%)	36 (69.2%)	140 (65.1%)	0.454
Without LTOT, n (%)	16 (28.6%)	14 (36.8%)	28 (41.2%)	16 (30.8%)	75 (34.9%)	0.454
Charlson Index, mean ± SD	3.1 ±1.8	2.9±2	2.9± 1.8	2.8±1.7	2.9± 1.8	0.285
Coronary heart disease, n (%)	31 (25.8%)	22 (24.4%)	25 (22.9%)	39 (36.1%)	117 (27.4%)	0.392
Length of stay, days, mean ± SD	11.0±7.8	10.0±6.5	11.5±8.7	10.3±8.1	10.7±7.8	0.587
Admissions/day, mean ± SD	1.3± 1.3	1.0±0.9	1.2±1.1	1.2± 1.1	1.2± 1.1	0.520
T_mean_, °C, mean ± SD	11.3±5.5	18.5±3.4	10.4±5.1	0.7±5.6	10.2±8.0	<0.001***
T_max_, °C, mean ± SD	16.2±6.6	23.2±4.3	14.3±6.3	3.1 ±5.5	14.2±9.2	<0.001***
T_min_, °C, mean ± SD	6.2±4.8	13.8±2.9	6.6±4.4	−2.0±6.2	6.2±7.3	<0.001***
ΔT, °C, mean ± SD	10.0±4.0	9.4±3.0	7.6±3.4	5.1 ±2.3	8.1 ±3.7	<0.001***
Air pressure, hPa, mean ± SD	1,009.9±9.9	1,008.8±5.0	1,007.9±7.7	1,011.7± 11.8	1,009.6±9.1	0.155
Air humidity, %, mean ± SD	64.4± 11.4	69.4±10.3	78.9±9.8	82.9±8.7	73.9±12.5	<0.001***

**Notes:**

* P<0.05;

*** *P*<0.001.

**Abbreviations:**BMI, body mass index; GOLD, global initiative for COPD; LTOT, long term oxygen therapy; T, temperature; SD, standard deviation.

**Table 2:** Comparison of AECOPD patient characteristics between hot and cold summers

Parameter	Hot summers	Cold summers	*P*-value
Admissions, n	250	313	–
Male, n (%)	129 (51.6%)	158 (50.5%)	0.800
Female, n (%)	121 (48.4%)	155 (49.5%)	0.800
Age, years, mean ± SD	70±11	71 ± 11	0.530
BMI, kg/m^2^, mean ± SD	25.3±6.6	25.6±6.4	0.611
Smoker, n (%)	82 (58.2%)	112 (47.1%)	0.016*
Former smoker, n (%)	47 (33.3%)	114 (47.9%)	0.016*
Non-smoker, n (%)	12 (8.5%)	12 (5.0%)	0.016*
Pack years, mean ± SD	51.1 ±32.6	48.8±26.3	0.858
FEV_1_, %, mean ± SD	39.7± 15.2	39.5± 15.0	0.852
GOLD I or II, n (%)	42 (24.4%)	54 (19.3%)	0.367
GOLD III, n (%)	80 (46.5%)	146 (52.1%)	0.367
GOLD IV, n (%)	50 (29.1%)	80 (28.6%)	0.367
With LTOT, n (%)	71 (82.6%)	125 (83.3%)	0.859
Without LTOT, n (%)	15 (17.4%)	25 (16.7%)	0.859
Charlson Index, mean ± SD	2.6± 1.8	3.1 ±2.0	0.002**
Length of stay, days, mean ± SD	8.3±5.1	8.3±6.0	0.353
Admissions/day, mean ± SD	1.4± 1.2	1.7± 1.3	0.006**
T_mean_, °C, mean ± SD	20.3±4.3	18.6±3.0	<0.001***
T_max_, °C, mean ± SD	25.4±5.2	23.3±4.0	<0.001***
T_min_, °C, mean ± SD	14.9±3.5	14.0±2.5	0.003**
ΔT, °C, mean ± SD	10.5±3.4	9.3±3.1	<0.001***
Air pressure, hPa, mean ± SD	1,009.3±6.1	1,008.0±5.5	0.059
Air humidity, %, mean ± SD	62.8± 14.3	67.8± 10.9	<0.001***

**Notes:**

* *P*<0.05;

** *P*<0.01;

*** *P*<0.001.

**Abbreviations:**BMI, body mass index; OLD, global initiative for COPD; LTOT, long term oxygen therapy; T, temperature; SD, standard deviation.

**Table 3:** Comparison of AECOPD patient comorbidities between hot and cold summers

Comorbidity	Hot summers	Cold summers	*P*-value
Coronary heart disease	83 (33.2%)	100 (31.9%)	0.786
Myocardial infarction	45 (17.6%)	33 (10.5%)	0.014*
Congestive heart failure	62 (24.8%)	100 (31.9%)	0.075
Peripheral vascular disease	19 (7.6%)	45 (14.4%)	0.016*
Cerebrovascular disease	21 (8.4%)	39 (12.5%)	0.132
Dementia	11 (4.4%)	20 (6.4%)	0.355
Chronic pulmonary disease^a^	250 (100%)	313 (100%)	–
Connective tissue disease	0 (0%)	0 (0%)	–
Ulcer disease	12 (4.8%)	13 (4.2%)	0.837
Renal disease	37 (14.8%)	65 (20.8%)	0.078
Liver disease	4 (1.6%)	12 (3.8%)	0.131
Diabetes	52 (20.8%)	85 (27.1%)	0.093
Hemiplegia	4 (1.6%)	6 (1.9%)	1.000
Any tumor	21 (8.4%)	53 (17.0%)	0.004**
Leukemia	2 (0.8%)	4 (1.3%)	0.698
Lymphoma	3 (1.2%)	1 (0.3%)	0.328
AIDS	0 (0%)	0 (0%)	-

**Notes:**

* *P*<0.05;

** *P*<0.01;

a Inclusion criterion.
